# Giant and Tunable Anisotropy of Nanoscale Friction in Graphene

**DOI:** 10.1038/srep31569

**Published:** 2016-08-18

**Authors:** Clara M. Almeida, Rodrigo Prioli, Benjamin Fragneaud, Luiz Gustavo Cançado, Ricardo Paupitz, Douglas S. Galvão, Marcelo De Cicco, Marcos G. Menezes, Carlos A. Achete, Rodrigo B. Capaz

**Affiliations:** 1 Divisão de Metrologia de Materiais, Instituto Nacional de Metrologia, Normalização e Qualidade Industrial (INMETRO), Campus Xerém, Av. Nossa Senhora das Graças 50, Xerém, Duque de Caxias, RJ, 25250-020, Brazil; 2 Departamento de Física, Pontifícia Universidade Católica do Rio de Janeiro, R. Marques de São Vicente 225, Rio de Janeiro, RJ, 22453-900, Brazil; 3 Departamento de Física, Instituto de Ciências Exatas, Cidade Universitária, Juiz de Fora, MG, 36036-900, Brazil; 4 Departamento de Física, Universidade Federal de Minas Gerais, Instituto de Ciências Exatas, Av. Antônio Carlos 6627, Belo Horizonte, MG, 31270-901, Brazil; 5 Departamento de Física, Universidade Estadual Paulista, Campus Rio Claro, Av. 24A 1515, Rio Claro, SP, 13506-900, Brazil; 6 Instituto de Física Gleb Wataghin, Universidade Estadual de Campinas, R. Sérgio Buarque de Holanda, 777, Cidade Universitária, Campinas, SP, 13083-859, Brazil; 7 Instituto de Física, Universidade Federal do Rio de Janeiro, Av. Athos da Silveira Ramos, 149-Cidade Universitária, Rio de Janeiro - RJ, 21941-590, Brazil

## Abstract

The nanoscale friction between an atomic force microscopy tip and graphene is investigated using friction force microscopy (FFM). During the tip movement, friction forces are observed to increase and then saturate in a highly anisotropic manner. As a result, the friction forces in graphene are highly dependent on the scanning direction: under some conditions, the energy dissipated along the armchair direction can be 80% higher than along the zigzag direction. In comparison, for highly-oriented pyrolitic graphite (HOPG), the friction anisotropy between armchair and zigzag directions is only 15%. This giant friction anisotropy in graphene results from anisotropies in the amplitudes of flexural deformations of the graphene sheet driven by the tip movement, not present in HOPG. The effect can be seen as a novel manifestation of the classical phenomenon of Euler buckling at the nanoscale, which provides the non-linear ingredients that amplify friction anisotropy. Simulations based on a novel version of the 2D Tomlinson model (modified to include the effects of flexural deformations), as well as fully atomistic molecular dynamics simulations and first-principles density-functional theory (DFT) calculations, are able to reproduce and explain the experimental observations.

The mechanical behavior of materials at the nanoscale may present completely novel and unexpected features as compared to their bulk counterparts. Particularly in the process of understanding and controlling friction at the nanoscale one faces new challenges and paradigm shifts^1^. Of special and significant interest is the topic of friction anisotropy at surfaces and nanostructures. Several experiments report large friction anisotropy in the sliding of two surfaces, and this has been attributed to the presence or absence of commensurability^2^,^3^. On the other hand, single-asperity atomic force microscopy (AFM) measurements are of different nature and they should be able to probe the intrinsic anisotropies of the surface structure on the atomic scale. These experiments have led to the observation of friction anisotropies on highly anisotropic crystal surfaces^4^,^5^ and compliant organic monolayers^6^.

The advent of single layer graphene opened up new possibilities for studies on the mechanisms of nanoscale friction. Major efforts have been addressed, for instance, to understand why friction increases as the number of atomic layers is reduced^7^,^8^,^9^,^10^. According to Lee *et al*.^8^, this effect originates from out-of-plane (flexural) elastic deformations, which become more prominent as the number of layers decreases, leading to puckering of the graphene sheets at the mechanical contact. This increases the surface-tip interaction, which in turn leads to higher friction forces.

One is then led to the question of friction anisotropy in graphene, which has been observed in exfoliated monolayer graphene, but with a 180° period angular pattern, and not the 60° period expected from the sixfold rotational symmetry of the graphene lattice^9^. Therefore, the previously observed anisotropy has been attributed to the out-of-plane elastic puckering resulting from differently oriented ripples in graphene. Sixfold friction anisotropies in highly oriented pyrolytic graphite (HOPG) have been observed and analyzed^11^,^12^,^13^,^14^,^15^, indicating that easy sliding directions are along zigzag, with higher friction forces along the armchair direction. However, friction anisotropy in graphite is small: only 15% higher along armchair with respect to zigzag, as we measure in this work.

In this work, we demonstrate that friction anisotropy in graphene is tunable by the normal force and can reach values as high as 80% (the friction is higher along the armchair direction), which represent a giant enhancement with respect to graphite. This behavior is quite unexpected, since the linear elastic properties of graphene (elastic constants, sound velocities, etc.) are isotropic, as expected from symmetry considerations. Using a combination of simulations based on the Prandtl-Tomlinson model^16^,^17^, fully atomistic molecular dynamics and density-functional theory (DFT), we explain this behavior as arising from the anisotropic amplification of tip-induced out-of-plane (flexural) deformations of the graphene sheet, not present in graphite. As we shall see, the effect can be seen as a novel manifestation of the classical phenomenon of Euler buckling at the nanoscale, which provides the non-linear ingredients that amplify friction anisotropy. These results represent a novel mechanism of energy dissipation in 2D systems, opening new possibilities for the design and control of nano-mechanical systems involving single layer materials.

Graphene flakes were prepared in ambient condition by micromechanical cleavage of bulk graphite. A typical flake used in our measurements is shown in Fig. 1a. All nanoscale friction measurements were performed in a friction force microscopy mode (FFM) in ambient air with 50% of relative humidity and at 20 °C. See Methods for details.

## Graphene crystallographic orientation

**Figure 1:**
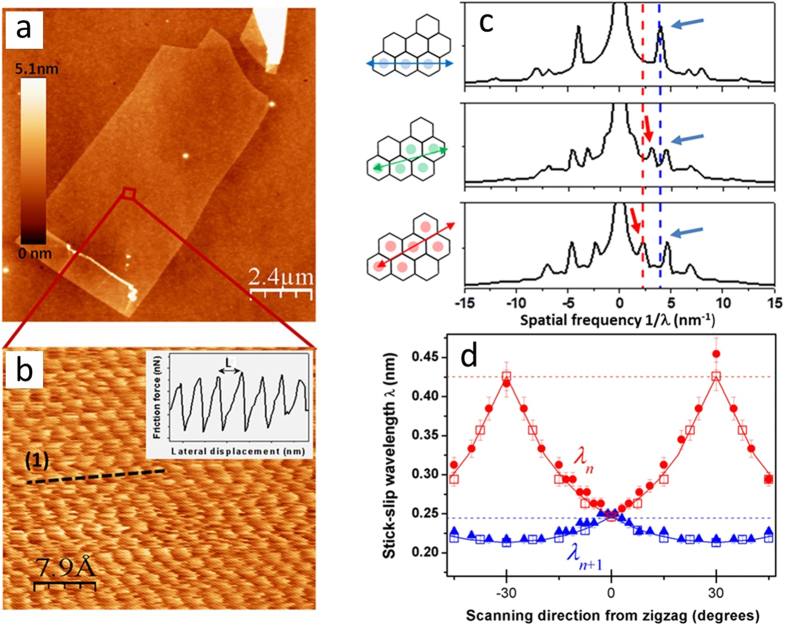
Crystallographic orientation of graphene flakes. Tapping mode AFM topography in (**a**) and lattice-resolution FFM image in (**b**) [obtained from the boxed area in a]. The inset in **b** shows an example of friction force profile as a function of lateral displacement of the tip taken along the dotted line drawn in the main panel. *L* is the length of the stick and slip jump. In (**c**), average FT spectra are presented for the zigzag (top); 15° misaligned with zigzag (middle); and armchair (bottom) crystallographic directions. Each spectrum is composed by a superposition of sharp peaks with a broad Lorentzian-like background. The peaks are related to the short-period stick-slip jumps and the background is associated with the slow spatial build-up of the friction forces caused by the gradual increase of the flexural deformations of the graphene sheet. The dashed blue and red vertical lines indicate expected values of spatial frequencies when the crystal is scanned along the zigzag (4.0 nm^−1^) and the armchair (2.3 nm^−1^) directions, respectively. On the left side of each panel we indicate the scanning direction and the nearby hexagon centers, as an illustration of tip trajectory, which is approximately a series of jumps between hexagon centers. In panel (**d**) the wavelengths associated to the main peaks in the FT spectra, marked with arrows in **c**, are plotted as a function of the angle between the tip scanning direction and the zigzag orientation.

Direct determination of the graphene’s crystallographic orientation is obtained via lattice resolution atomic force microscopy images^18^. Figure 1b shows an FFM image of monolayer graphene. As depicted in the image, the AFM tip moves in a discontinuous way on top of the graphene layer following a stick and slip pattern. Although weak van der Waals forces are responsible for the adhesion of the graphene sheet to the substrate, we have not observe any displacement of the graphene with respect to the substrate during scanning, possibly because of the large sizes of our graphene flakes (which result in large contact areas between graphene and substrate). In the inset, the profile taken along the dotted line in the main panel can be observed. Similar profiles obtained along several crystallographic orientations reveal stick and slip events with various jump distances *L* as illustrated in the inset of Fig. 1b. In order to analyze the periodicity of stick and slip events, a Fourier Transform (FT) processing was performed at each scan line of the friction image. Several images were acquired for each scanning direction and Fig. 1c presents the average FT spectra for the zigzag (i); 15° rotated with respect to the zigzag (ii); and armchair (iii) crystallographic directions (additional spectra for other directions are shown in the Supplementary Information). The dashed blue and red vertical lines represent the values of spatial frequencies associated with the zigzag (4.0 nm^−1^) and armchair (2.3 nm^−1^) lattice periodicities, respectively (we have considered the graphene lattice constant to be *a* = 0.246 nm). When the tip scans along the zigzag crystallographic direction (30^°^), a single prominent peak at 4.0 nm^−1^ is clearly observed in the FT spectrum. On the other hand, when the tip scans along any other direction, at least two prominent peaks are observed in the FT spectra. These two features have spatial frequencies of 1/*λ*_*n*_ and 1/*λ*_*n*+1_, and the largest separation between them occurs for scanning along the armchair direction (±30°). Figure 1d shows the corresponding wavelengths *λ*_*n*_ and *λ*_*n*+1_ as a function of the scanning angle (with respect to the zigzag direction): full circles and triangles are experimental data, empty squares are results from Prandtl-Tomlinson simulations, and the solid lines represent an analytical approximation (see Supplementary Information available), which gives 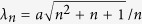 and 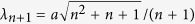, with 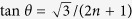. The agreement is excellent between the various approaches. Following this procedure, it is possible to determine the crystallographic orientation of the observed monolayer graphene with an accuracy of ≈2°. This procedure is considerably more precise than a previously presented one^18^.

## Friction anisotropy

**Figure 2:**
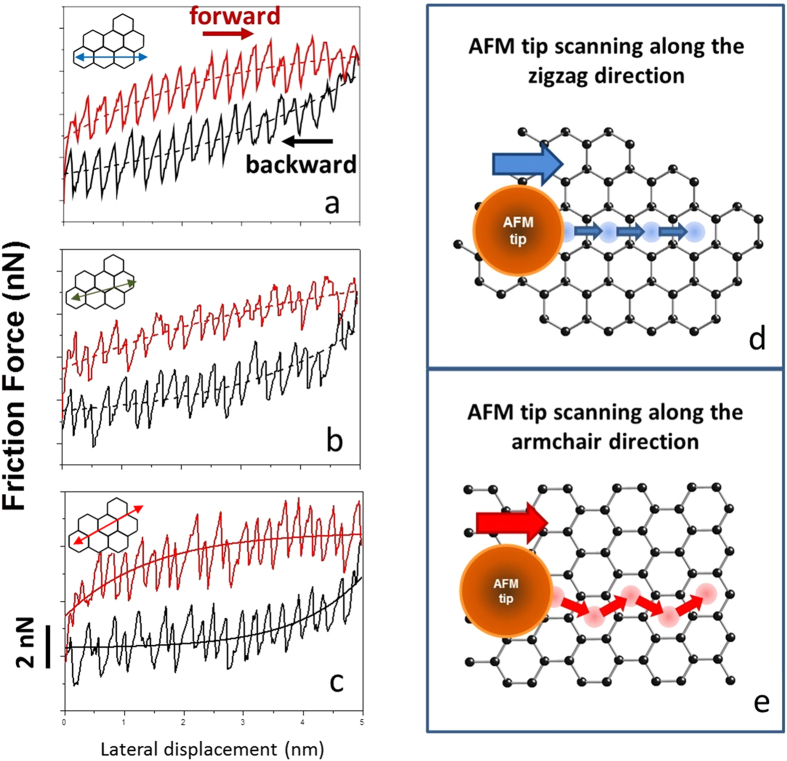
Friction forces in graphene. (**a,b,c**) show friction force values as a function of the tip lateral displacement along the zigzag (**a**), 15° off zigzag (**b**), and armchair (**c**) directions. Dashed lines are exponentially-saturating functions illustrating the strengthening and saturation of friction forces. Incidentally, Fourier transforms of exponential functions are Lorentzians, which explains the Lorentzian background of FT spectra shown in Fig. 1. Insets illustrate tip scanning directions over the graphene sheet. Figure (**d**,**e**) illustrate the spatial distribution of local minima in the tip-graphene interaction potential along the zigzag and armchair directions, respectively. Blue and red circles inside the hexagonal lattice of graphene represent sticking points at local potential minima for zigzag and armchair directions, respectively, and arrows indicate slip jumps. Along the zigzag direction, all jumps are equal, giving the corresponding force profile the appearance of a simple sawtooth function. Along the armchair direction, jumps form a zigzag pattern, which gives the correspondent force profile a double-period structure. Along arbitrary directions, force profiles are more complicated and discussed in detail in the Supplementary Information.

Once we have determined the crystallographic orientation of the graphene sheet, friction forces can be measured along specific directions. Figure 2a–c show friction force profile loops (forward and backward movement), obtained when the tip is scanned along zigzag (a), misaligned by 15^°^ with respect to zigzag (b), and armchair (c) directions. All profiles present short-period stick and slip jumps superimposed with slow build-up of friction forces, eventually reaching saturation after the tip completes approximately 10 stick-slip cycles. The orientation dependence of the stick-slip pattern^11^ is discussed in detail in the Figure caption and Supplementary Information. We associate the slow build-up of friction forces (also called “tilted loops”), illustrated by the dashed lines in Fig. 2a–c, with a “strengthening” of the tip-surface interaction due to increasing flexural deformations of the monolayer as the tip moves: puckering of graphene around the tip enhances the tip-graphene interaction, which in turn increases the work needed to move the tip^8^. Similar FFM measurements performed in the HOPG surface provide significantly smaller friction forces, and no tilt in the friction loops is observed (Supplementary Information).

**Figure 3:**
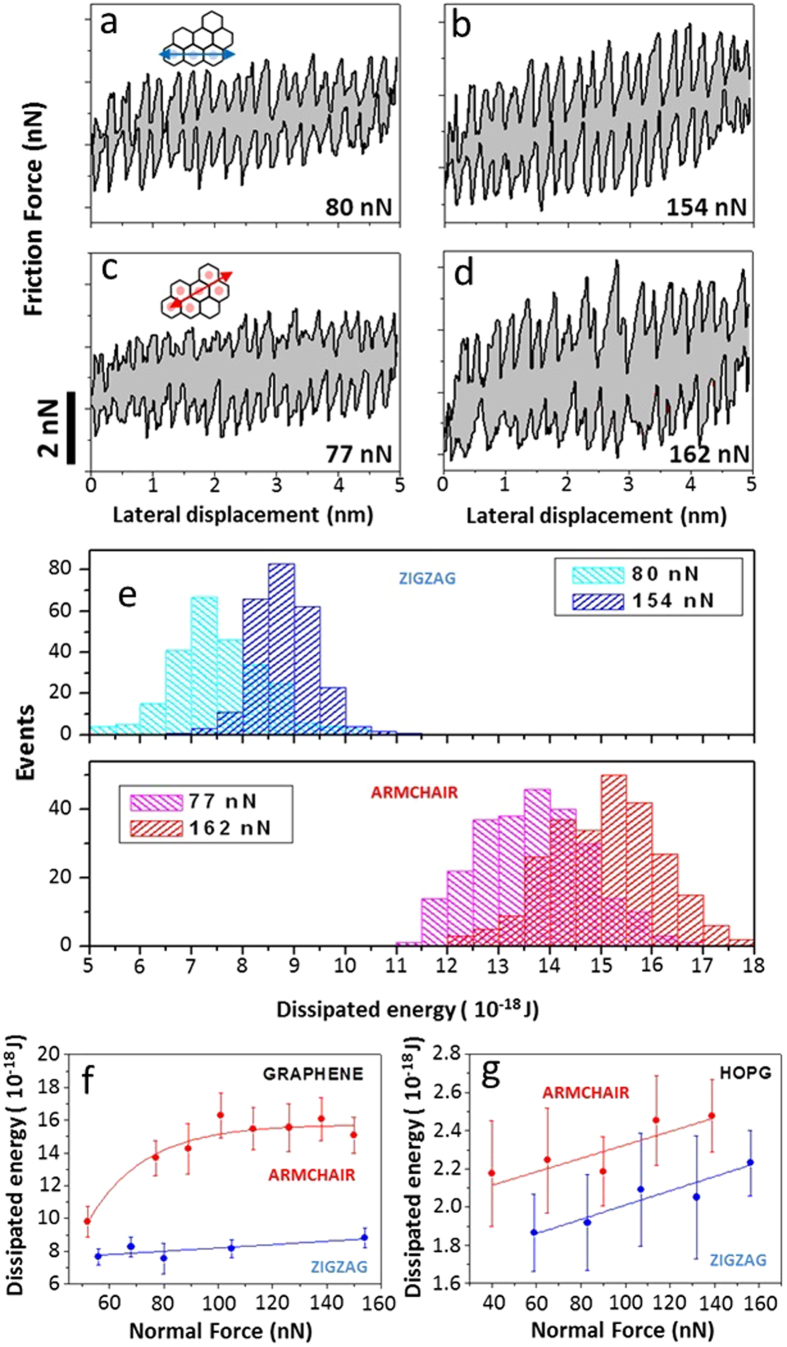
Energy dissipation during friction loops. Friction loops along zigzag (**a,b**), and armchair (**c,d**) scanning directions for different normal forces: (**a**) 80 nN, (**b**) 154 nN, (**c**) 77 nN and (**d**) 162 nN. The gray area represents the amount of energy dissipated during the friction process. (**e**) Histograms of the dissipated energy while scanning the AFM tip along the zigzag (top) and armchair (bottom) directions for two different normal forces. Dissipated energy as a function of normal force for graphene (**f**) and HOPG (**g**), for both armchair (red) and zigzag (blue) directions respectively.

Strengthening of tip-surface interaction induced by the tip motion certainly increases energy dissipation, by converting kinetic energy of the tip into lattice vibration energy. The energy dissipated in each scanning cycle is obtained by integrating the friction force over the lateral displacement in the forward and backwards scan loops, as illustrated in Fig. 3 for the zigzag (a,b) and armchair (c,d) directions at different applied normal forces. In Fig. 3e, statistical analyses of the dissipated energy along the zigzag (top) and armchair (bottom) directions are presented for two different values (~80 nN and ~150 nN) of normal force (F_N_). Surprisingly, the energy dissipated along the armchair direction is about 80% larger than the energy dissipated along the zigzag direction, for large normal forces. Interestingly, this giant friction anisotropy can be tuned by the applied normal force: friction enhancement along armchair with respect to zigzag increases from roughly 15% to reaching saturation at ~80% as the normal force increases from 50 nN to 150 nN as shown in Fig. 3f. Further increase in the applied normal force may lead to additional ripples in graphene and plastic deformation of the scanning tip (see supplemental material). By comparison, friction anisotropy in HOPG is considerably smaller, roughly 15% larger along armchair and nearly independent of normal forces, as it can be seen in Fig. 3g. In fact, this small friction anisotropy can be understood in a very simple manner, by counting the number of stick-slip jumps per unit distance, which are roughly 15% larger in the armchair direction as compared to zigzag (see Supplementary Information).

Friction anisotropy has been previously observed in bulk graphite, and was associated with the incommensurability of an “effective tip” generated by adhesion of a small flake of graphite to the tip apex^3^. In this case, the lattice mismatch between the effective tip and the graphite surface can be strongly influenced by the scanning direction, leading to the observed anisotropy in the friction forces. However, such lattice commensurability arguments do not apply to our experiments, since we have an amorphous Si_3_N_4_ tip scanning over a single graphene layer. The anisotropy in HOPG observed in our experiments has atomic-scale origin and it is discussed in detail in the Supplementary Information available.

As it is evident from the comparison to the HOPG case, the giant and tunable anisotropy observed in graphene must be related to flexural deformations of the monolayer, which are likely enhanced when the tip moves along the armchair direction, with respect to the zigzag. This is surprising, because linear elastic constants are isotropic in graphene, so one should not expect to be any easier to buckle a graphene sheet by applying forces along any specific direction. Besides, assuming that the graphene sheet is flat when the motion begins, lateral forces exerted by the tip on the sheet are only 15% larger along armchair (the friction anisotropy of HOPG), therefore there must be some sort of nonlinear ingredient responsible to amplify the anisotropy of flexural deformations as the tip moves.

## Modified Prandtl-Tomlinson model

**Figure 4:**
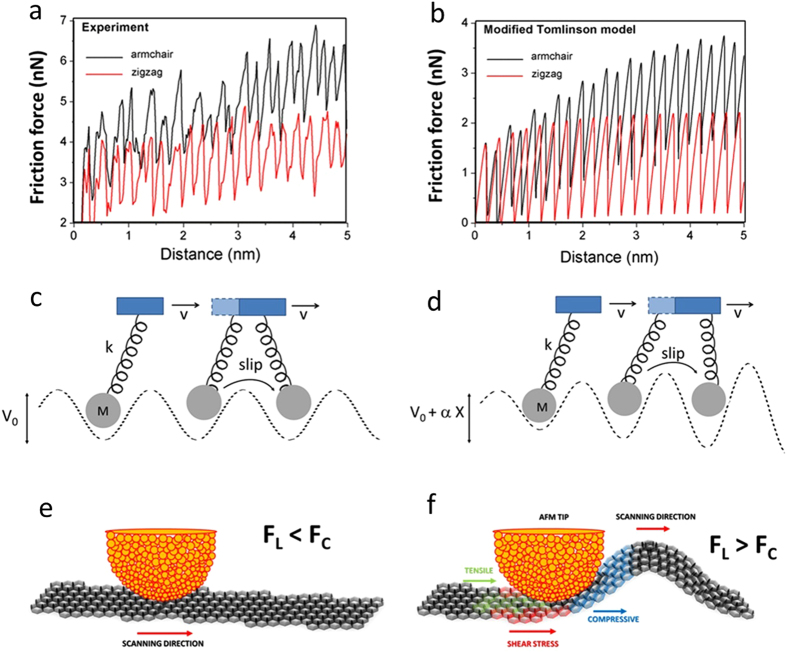
Friction force profiles. Experimental (**a**) and simulated (**b**) friction force profiles. In both cases, large enhancement of friction forces occurs for the armchair direction during the transient build-up regime, in comparison to the zigzag direction. This illustrates how giant anisotropy amplification is reproduced by the modified Tomlinson model simulations. (**c**) Illustration of the basic stick-slip mechanisms of the usual Tomlinson model. (**d**) In the modified Tomlinson model, the tip-surface interaction increases with scanning distance because of flexural deformations, thus increasing lateral forces and friction. (**e**) When lateral forces *F*_*L*_ are smaller than the critical force *F*_*c*_ for Euler buckling, the graphene sheet remains planar, (**d**) in the opposite situation, flexural deformations (buckling) occurs.

We are able to model and understand the giant friction anisotropy in a graphene using a modified version of the well-stablished Prandtl-Tomlinson model^16^,^17^. In the usual model for HOPG, illustrated in Fig. 4c, the tip apex is modeled by a particle with mass *m* and position **r**_*t*_, interacting with the surface via a potential energy *V*(**r**_*t*_). For HOPG, we use^13^:


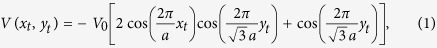


with *a* = 0.246 nm. This potential energy corresponds to placing the zigzag direction along *x*. The base of the microscope is located at position **r**_*M*_, and the elastic energy of the strained tip is modeled by an isotropic harmonic potential: 
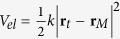
. The base moves at constant speed and the tip dynamics is modeled by a Langevin equation:


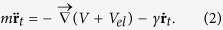


The stick-and-slip motion of the tip apex gives rise to friction and the component *F*_*t*_ of the elastic force along the base trajectory is integrated along the path to give the dissipated energy. Technical details of the simulations are presented in the Supplementary Information. This simple model reproduces the small (15%) friction enhancement along the armchair direction with respect to zigzag for HOPG.

We then introduce flexural degrees of freedom of graphene by adding an effective dynamical variable *X* to describe flexural displacements, which evolves according to the following equation of motion:


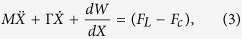


where *M* is an effective mass that describes the inert, interacting with the surface via a potential energyrtia of the flexural mode and *W*(*X*) is the restoring potential of the flexural mode, in which we include a non-harmonic cubic component on the potential 
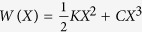
, with *C* > 0. The cubic term is important to make the system stable after we introduce the feedback mechanism by which the increase in flexural amplitude enhances the tip-surface interaction, to be discussed below. Damping is introduced by the Γ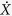 term, and again we assume critical damping 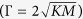. *F*_*L*_ is the lateral force due to the tip, which drives surface buckling only if it becomes larger than a critical force *F*_*c*_. This is the well-known condition for Euler buckling which occurs not only for macroscopic rods and membranes^19^,^20^ but also in related nanoscale objects^21^,^22^.

A feedback mechanism by which the increase in flexural amplitude enhances the tip-surface interaction is introduced simply by adding a linear dependence of the tip-surface potential energy amplitude *V*_0_ on the flexural amplitude *X*: *V*_0_→*V*_0_ + *αX*. This is illustrated schematically in Fig. 4d. We then evolve simultaneously both the tip and flexural mode dynamics using a Verlet algorithm^23^. For a certain range of parameters, the combined dynamics reproduces well the observed amplification in friction anisotropy observed experimentally, as shown in Fig. 4. Clearly, during the transient build-up of friction forces, anisotropy enhances by roughly a factor two for both experiments and simulations. We emphasize that, in order to reproduce the effect, the elastic properties of the sheet are not required to be anisotropic. In fact, first-principles calculations based on DFT show that critical forces for Euler buckling, as well as buckling amplitudes, are quite independent on the direction of the applied force (see Supplementary Information). The critical forces and stresses applied by the tip might induce elastic strain in the substrate as well; however, the buckling is induced solely in the graphene sheet. Moreover, since the substrate is amorphous SiO_2_, it should have no effect on friction anisotropy. The anisotropy amplification effect can be easily understood by considering an extreme situation in which the lateral forces along armchair, initially only 15% larger than zigzag ones when the sheet is planar (as for the HOPG case), become larger than the critical buckling force as the tip moves, whereas zigzag forces do not. In this special case, armchair scanning causes buckling and zigzag scanning does not, leading to giant anisotropy amplification. The effect can be tuned by increasing the normal forces, which in turn increase the lateral friction forces.

## Molecular dynamics and stress induced by the scanning tip

**Figure 5:**
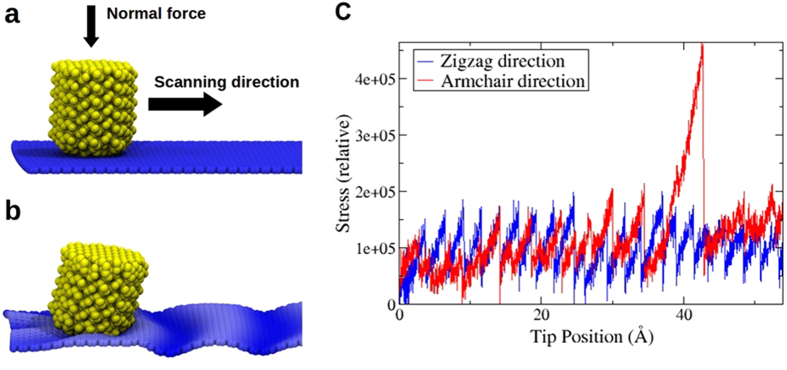
Atomistic model for MD simulations: (**a**) A model AFM tip (yellow spheres) is pushed against (down towards) a graphene layer (blue atoms) and scans the surface laterally. The graphene layer interacts via van der Waals with a substrate below it (not shown). (**b**) Snapshot of a MD simulation showing the corrugation pattern of the graphene layer caused by the AFM tip scan. (**c**) Relative von Mises stress as a function of distance, showing clearly the stick-slip pattern and the larger stress enhancement for armchair scanning with respect to zigzag during the transient regime, for the same conditions of normal force. One can even notice a double stick-slip event near 40 Å for the armchair direction, in which the stress build-up reaches substantially larger values than typical single stick-slip events.

Finally, in order to provide an atomistic visualization of the mechanisms leading to these phenomena, we perform classical molecular dynamics (MD) simulations. Our simulation model is composed of an elastic tip (the AFM tip) and a single layer of graphene, which interacts via van der Waals forces with a horizontal substrate which is held fixed below it. The AFM tip is pushed against the surface using a constant force (~0.3 pN) perpendicular to the graphene plane and forced to move along the horizontal direction, parallel to the membrane, as illustrated in Fig. 5a. Technical details and movies of the simulations are provided in the Supplementary Information. Similarly to the Tomlinson model simulations, there is initially a transient regime in which, superimposed to stick-slip events, gradual stress build-up takes place, until a dynamical steady-state is reached. A convenient way to measure the overall stress in the system is through a scalar quantity *σ*_*vm*_ known as the von Mises stress^24^,^25^, defined from the virial stress tensor^26^ components *σ*_*ij*_ as:


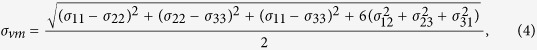


Fig. 5b shows a snapshot of a particular MD simulation, where the corrugation pattern induced in the graphene by the AFM tip motion is clearly seen. Figure 5c shows the evolution of the von Mises stress with the distance traveled by the tip. Interestingly, the stick-slip sawtooth pattern is also clearly seen in the von Mises stress. It is also evident a larger stress enhancement for armchair scanning with respect to zigzag during the transient regime, considering the same conditions of normal force.

## Conclusions

In summary, we demonstrate the existence of a giant and tunable anisotropy in nanoscale friction between an AFM tip and graphene. The microscope tip was observed to move in a discontinuous stick and slip mode allowing the identification of the graphene crystallographic directions. By increasing the normal force, the anisotropy increases from 15% (same as HOPG) to roughly 80% larger for scanning along the armchair direction, with respect to the zigzag one. The nonlinear mechanism responsible for amplifying the anisotropy is related to Euler buckling in membranes, which leads to flexural deformations of the graphene sheet, causing puckering of graphene around the tip and enhancing tip-surface interactions, in a feedback mechanism. The proposed mechanism is supported by theoretical calculations in three levels: density functional theory, molecular dynamics simulations and Tomlinson model. This novel behavior is an important step in understanding and controlling friction in single-atom-thick membrane at the nanoscale.

## Methods

### Sample preparation

Graphene flakes were prepared in ambient conditions by micromechanical cleavage of bulk graphite onto a 300 nm SiO_2_ layer on top of a Si substrate^27^. The samples were examined by optical microscopy and tapping mode atomic force microscopy. The number of layers in the graphene flakes was confirmed by Raman spectroscopy^28^.

### Friction force measurements

All nanoscale friction measurements showing atomic-scale lattice resolution were acquired using silicon nitride V-shape cantilevers, with tip radii of about 20 nm and calibrated normal and lateral spring constants of 0.40 ± 0.01 N/m and 85.4 ± 4.2 N/m respectively. During quantitative FFM measurements, the scanning direction was kept perpendicular to the cantilever main axis in such a way that the friction force experienced by the tip was measured by the lateral torsion of the cantilever. The microscope was calibrated for each tip used, and the lateral force was obtained from the product between the cantilever’s lateral spring constant and the lateral tangential displacement of the tip measured in the position sensitivity photodetector^29^. The microscope gains were set close to zero, and the scans were performed at 150 nm/s.

## Additional Information

**How to cite this article**: Almeida, C. M. *et al*. Giant and Tunable Anisotropy of Nanoscale Friction in Graphene. *Sci. Rep.* **6**, 31569; doi: 10.1038/srep31569 (2016).

## Supplementary Material

Supplementary Information
