# Supplementary material for: Giant and Tunable Anisotropy of Nanoscale Friction in Graphene
Source: Sci Rep. 2016 Aug 18;6:31569. doi: 10.1038/srep31569 (PMC4989147; doi:10.1038/srep31569)
Supplement: Supplementary Information [file srep31569-s1.pdf]

Supplementary information

Giant and Tunable Anisotropy of Nanoscale Friction in Graphene

Clara M. Almeida[1], Rodrigo Prioli[2], Benjamin Fragneaud[1,3], Luiz Gustavo Cançado[1,4], Ricardo Paupitz[5], Douglas S. Galvão[6], Marcelo de Cicco[1], Marcos G. Menezes[7], Carlos A. Achete[1], and Rodrigo B. Capaz[1,7]*

[1] Divisão de Metrologia de Materiais, Instituto Nacional de Metrologia, Normalização e Qualidade Industrial (INMETRO), Campus Xerém, Av. Nossa Senhora das Graças 50, Xerém, Duque de Caxias, RJ, 25250-020, Brazil.

[2] Departamento de Física, Pontifícia Universidade Católica do Rio de Janeiro, R. Marques de São Vicente 225, Rio de Janeiro, RJ, 22453-900, Brazil.

[3] Departamento de Física, Instituto de Ciências Exatas, Cidade Universitária, Juiz de Fora, MG, 36036-900, Brazil.

[4] Departamento de Física, Universidade Federal de Minas Gerais, Instituto de Ciências Exatas, Av. Antônio Carlos 6627, Belo Horizonte, MG, 31270-901, Brazil.

[5] Departamento de Física, Universidade Estadual Paulista, Campus Rio Claro, Av. 24A 1515, Rio Claro, SP, 13506-900, Brazil.

[6] Instituto de Física Gleb Wataghin, Universidade Estadual de Campinas, R. Sérgio Buarque de Holanda, 777, Cidade Universitária, Campinas, SP, 13083-859, Brazil.

[7] Instituto de Física, Universidade Federal do Rio de Janeiro, Av. Athos da Silveira Ramos, 149 - Cidade Universitária, Rio de Janeiro - RJ, 21941-590, Brazil.

Fourier Transform spectra for additional crystallographic orientations

Friction force microscopy images were acquired for several scanning directions in order to analyze the periodicity of stick and slip events as a function of the scanning angle. The Fourier Transform analysis was performed at each scan line of the friction images and the average spectrum for each direction is shown in Fig. S1. Starting from the zigzag (on top), each spectrum was obtained from scans rotated by an additional ~ 5° from the previous ones.

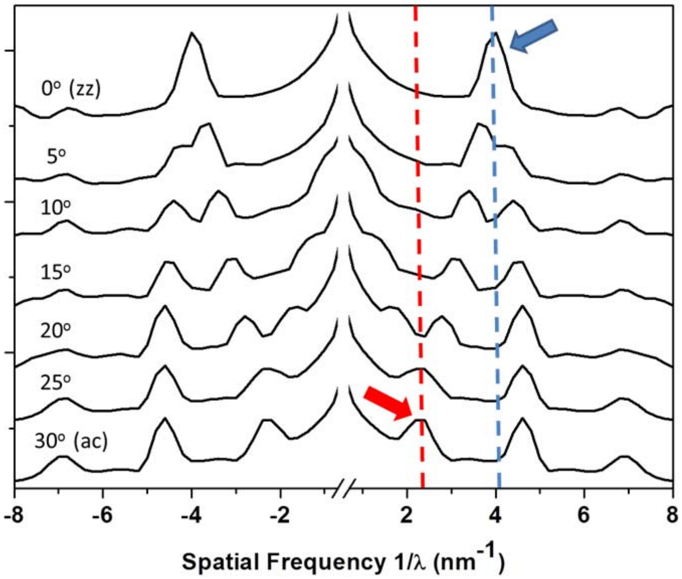

Figure S1. FT spectra for additional crystallographic directions. Average FT spectra are presented for several scanning directions between the zigzag (top) and armchair (bottom) crystallographic directions. The values of the spatial frequencies associated with the zigzag (4.0 nm^{-1}) and armchair (2.3 nm^{-1}) lattice periodicities are highlighted by the dashed blue and red vertical lines and arrows respectively.

As the AFM tip scans along the zigzag crystallographic direction, a single peak (at 4.0 nm^{-1}) is observed in the FT spectrum. As the scanning direction is varied, two

prominent peaks are observed in the FT spectra. The separation between these two peaks increases as the scanning direction is deviated from the zigzag.

Modeling the angle dependence of the Fourier spectra of lateral force profiles

The angle dependence of the main peaks observed in both experimental and simulated Fourier spectra can be understood by a simple model. Let us consider first the simpler cases of zigzag (θ = 0°) and armchair (θ = 30°) directions. For the zigzag direction, the tip develops a nearly straight stick-slip motion with a spatial period equal to graphene lattice constant *a* (Fig. S2a). The force profile resembles a sawtooth wave, so the corresponding Fourier spectrum will be composed of main peaks at $\lambda_{\pm 1}^{-1} = \pm 1/a$ and smaller peaks at higher harmonics $\lambda_{\pm m}^{-1} = \pm m/a$, m = 2, 3, 4,... (Fig. S2a). For armchair scans, the tip develops a zigzag stick-slip motion, as it jumps between nearest-neighbors hexagon centers (Fig. S2c). The spatial period of such movement (projected along the scanning direction) is approximately $\lambda_2 = \sqrt{3}a/2$, therefore the main Fourier peaks will occur at $\lambda_{\pm 2}^{-1} = \pm 2/\left(\sqrt{3}a\right)$ (and smaller peaks at higher harmonics). However, in general the two jumps in a given zigzag will not be symmetric with respect to the scanning trajectory (dashed lines in Fig. S5e), so the corresponding force profile will have a larger period modulation of $\lambda_1 = \sqrt{3}a$. This gives rise to peaks at $\lambda_{\pm 1}^{-1} = \pm 1/\left(\sqrt{3}a\right)$ in the corresponding Fourier spectrum. This is consistent with the fact that the full period of the tip movement along the armchair direction is $\lambda_1 = \sqrt{3}a$.

In other to analyze the tip motion for a general scan angle θ, it is instructive to consider directions that give rise to commensurate (periodic) motions in the underlying graphene lattice. It is also instructive to consider the near-zigzag (small θ) limit. In this case, it is interesting to make a correspondence between the spatial period of the

scanning movement and the chiral vector in a (n,1) single-wall carbon nanotube[S1]. In this particular situation, as illustrated in Fig. S2b for the case n = 4, the tip will likely move in a sequence of n jumps along the $\vec{a}_1$ primitive vector of the graphene lattice, followed by 1 jump along $\vec{a}_2$. The projections along the scanning direction for the two types of jump will be $L_1 = a\cos\theta$ and $L_2 = a\cos(60^o - \theta)$, respectively, so the force profiles in real space will display approximately a sequence of n jumps of size L_1 and one jump of size L_2. However, the full period of the tip motion will be given by the magnitude of the chiral vector $\lambda_1 = a\sqrt{n^2 + n + 1}$, so the Fourier peaks must occur at $\lambda_{\pm m}^{-1} = \pm m/\lambda_1$. Interestingly, for this particular wave, the highest Fourier peak will not correspond to the fundamental mode m = ±1, but rather to the higher harmonics $m = \pm(n+1)$ and $m = \pm n$, since in these cases, for large n, $m/\lambda_1 \approx 1/a$ and the sawtooth wave with jumps along $\vec{a}_1$ will be nearly resonant with the sinusoidal Fourier waves of wavevectors $\lambda_n^{-1} = n/\lambda_1$ and $\lambda_{n+1}^{-1} = (n+1)/\lambda_1$, as shown in Fig. S2b. This gives rise to the two-peak structure observed in the Fourier spectra for scanning along θ. The corresponding wavelengths $\lambda_n = \lambda_1/n$ and $\lambda_{n+1} = \lambda_1/(n+1)$ are plotted as a function of θ in Fig. 1d of the main text. Interestingly, the model describes extremely well the experimental and simulated peak positions, even in situations in which the scanning is not necessarily commensurate with the graphene lattice nor in the near-zigzag limit.

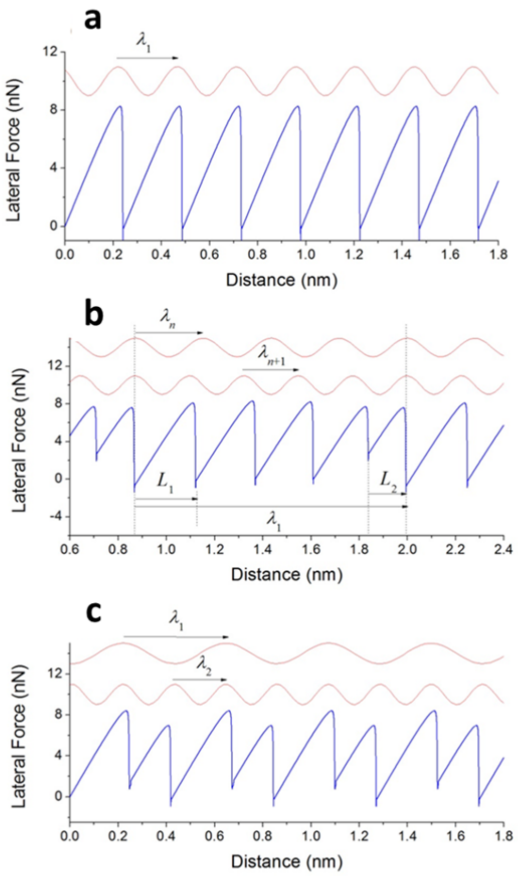

Figure S2. Angle dependence of the Fourier spectra of lateral force profiles. Real space profiles (blue) and main Fourier components (red) of lateral forces as a function of distance, for (a) zigzag, (b) $\theta \approx 10.9^{o}$, (c) armchair directions

Friction loops and anisotropy in HOPG

FFM measurements as a function of the crystallographic orientation were also performed in highly oriented pyrolitic graphite (HOPG). The images show the discontinuous movement of the tip following the stick and slip pattern, similar to those obtained in graphene. An important difference, however, is the absence of build-up of lateral forces during scan ("tilted loops"). Figures S3a and S3b show the forward and

backward friction force profiles, forming friction loops, obtained when the tip was scanned along the zigzag (a) and along the armchair direction (c) in HOPG.

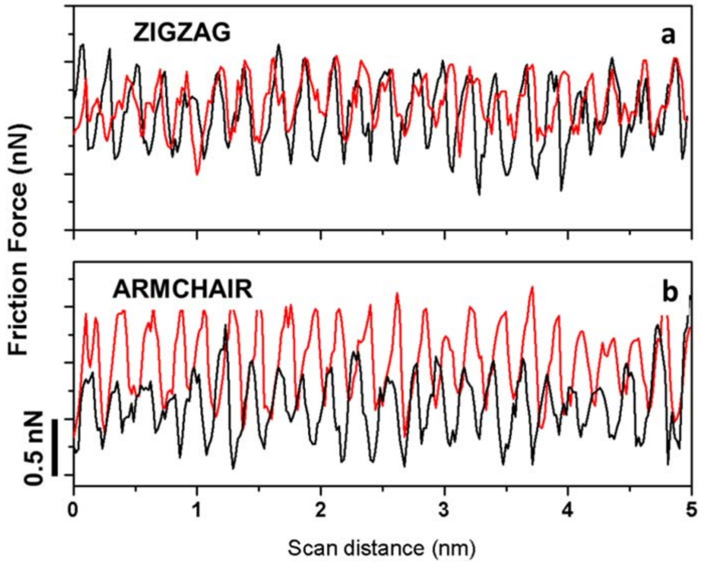

Figure S3. Friction loops in HOPG. Friction force as a function of scan distance along (a) zizgzag and (b) armchair directions. Red corresponds to forward scan and black corresponds to backward scan.

Friction in the HOPG was found to be significantly smaller than for monolayer graphene. Since the interaction between the sheets in HOPG is stronger than the interaction between a monolayer graphene and the SiO_2 substrate, when the tip makes contact with the graphite surface, no puckering around the tip occurs and no wrinkle is formed at the front face of the moving tip while scanning, resulting in non-tilted force friction loops.

Energy dissipation in HOPG was obtained by integrating the friction force over the forward and backwards scans. Figure S4 shows the statistical analysis of the

dissipated energy of graphite scanned along the zigzag (a) and armchair (c) directions at different applied normal forces. The energy dissipated along the armchair direction is ~ 15% higher than along the zigzag crystallographic direction, for different values of applied normal force.

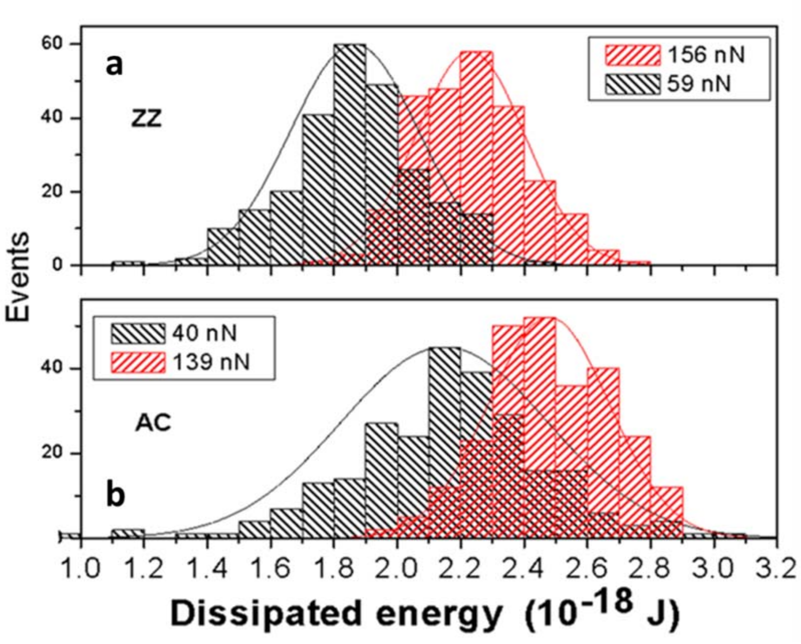

Figure S4. Energy dissipation in HOPG. Histograms showing the dissipated energy in graphite while the tip was scanned along the (a) zigzag with normal force of 59 nN and 156 nN; and along the (b) armchair direction with normal force of 40 nN and 139 nN.

Tomlinson model simulations for HOPG

For the Tomlinson model[S2,S3] used to simulate AFM scans in HOPG, described in Equations (1) and (2) of the main text, we adopt the following parameters: $V_0 = 0.22$ eV, $m = 10^{-8}$ kg, $k = 12.5$ N/m, $\gamma = 7\times10^{-4}$ N.s/m[S4]. The tip trajectory is integrated using a velocity Verlet algorithm, with a time step of 2 μs[S5]. The AFM tip base moves at constant speed of 40 nm/s.

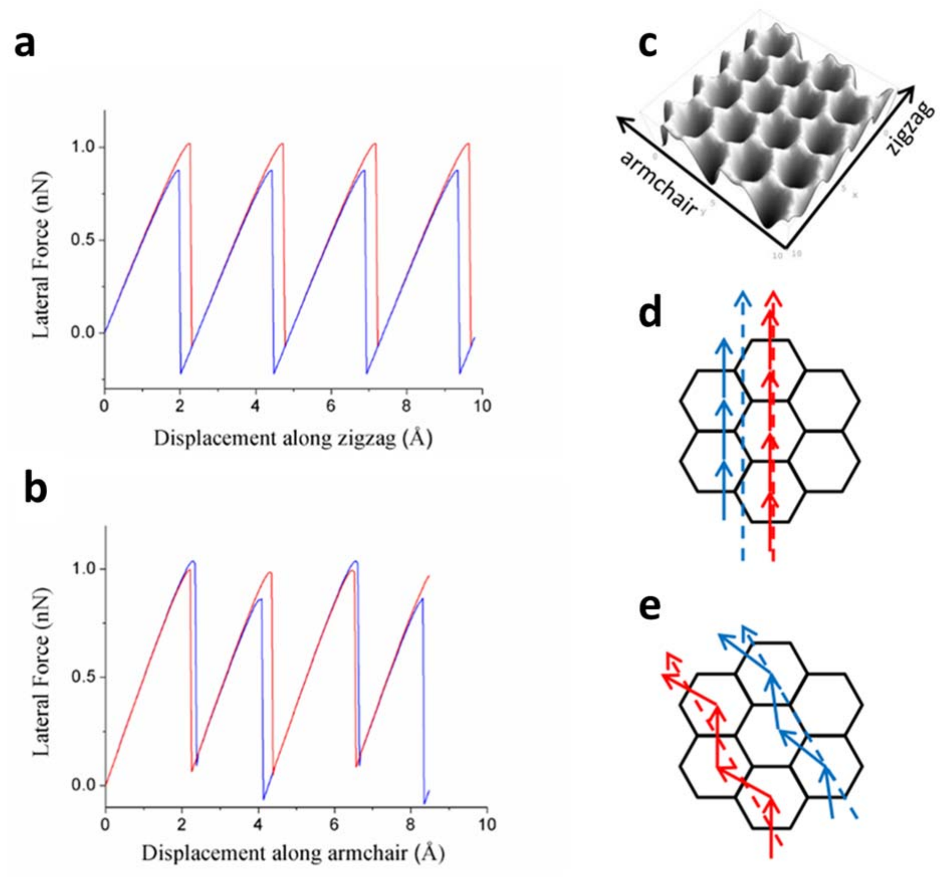

Figure S5. Tomlinson model simulations for HOPG. (a) Zigzag and (b) armchair lateral force profiles for different tip base trajectories. (c) Tip-surface interaction potential energy plot. (d) Zigzag and (e) armchair schematic tip base trajectories (dashed arrows) stick-slip tip apex jumps (full arrows). There is a color correspondence (red and blue) between the profiles in Figs. (a) and (b) and the trajectories in Figs. (d) and (e).

Fig. S5a and S5b show simulated lateral force profiles for zigzag and armchair scans, respectively. Blue and red curves correspond to different choices of the initial position of the AFM tip base, as indicated by the same colors in Figs. S5d and S5e. As it turns out, there are infinitely many possible trajectories for a given scan direction, corresponding to different initial positions of the tip base. Dashed arrows correspond to the tip base trajectory and full arrows indicate schematically the tip apex jumps, as it

tries to stay as close as possible to hexagon centers, which correspond to the minima of tip-surface interaction potential, shown in Fig. S5c. We should stress that, for zigzag scans, all trajectories lead to a single-period sawtooth-like force profile, as shown in Fig. S5a. However, for armchair scans, most trajectories lead to a double-period structure, as shown by the blue curve in Fig. S5b. Only very special trajectories, such as the red one in Fig. S5e, lead to single-period force profiles.

To obtain the dissipated energy for each scan direction, we perform an average over all possible tip starting positions. This results in an energy dissipation per distance of $\varepsilon_{zz} = 2.66$ eV/nm in the zigzag direction and $\varepsilon_{ac} = 3.01$ eV/nm in the armchair direction, resulting in a 13.2% anisotropy, very close to our experimental result of 15%. We use the Tomlinson model to obtain the energy dissipation along intermediate directions between zigzag and armchair, as shown by the squares in Fig. S6. The blue line is an interpolation function $\varepsilon(\theta) = \varepsilon_{zz} + (\varepsilon_{ac} - \varepsilon_{zz})\sin(3\theta)$.

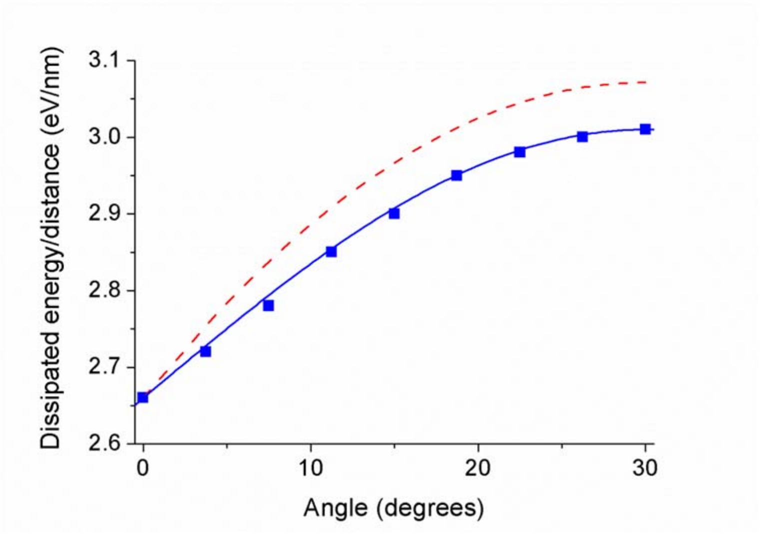

Figure S6. Dissipated energy in HOPG. Dissipated energy per unit distance as a function of scan angle with respect to zigzag. Blue squares are the results of Tomlinson model simulations, the blue line is a fit using $\varepsilon(\theta) = \varepsilon_{zz} + (\varepsilon_{ac} - \varepsilon_{zz})\sin(3\theta)$, and the dashed red line is a simple model based on the geometry of stick-slip jumps (described in the text).

The red dashed line in Fig. S6 is a simple model based on the geometry of the stick-slip tip dynamics at the HOPG surface. It is assumed that the dissipated energy is proportional to the number of stick-slip jumps, i.e., every jump contributes equally to the dissipated energy. It is further assumed that the tip apex jumps from an hexagon center to the next. Using these assumptions, it is clear from Figs. S5d and S5e that the ratio between armchair and zigzag energy dissipation must be $2/\sqrt{3} \approx 1.15$, amazingly close to the experimentally measured 15% friction enhancement for the armchair direction with respect to zigzag, As it turns out, using the same geometrical argument, we can determine the relative energy dissipation for any scan direction with respect to zigzag. In fact, the geometry is quite analogous to that of single-wall carbon nanotubes (SWNTs)[S5]: in analogy to SWNT chiral indices (n,m), the tip appex makes $n + m$ jumps when the tip base scans along an angle θ with respect to zigzag, which plays the role of the chiral angle in SWNTs, with $\tan\theta = \sqrt{3}m/(2n+m)$. The distance traveled by the tip base plays the role of SWNT circumference $\sqrt{n^2+nm+m^2}$ (in units of the graphene lattice constant). The red dashed line then shows the quantity $(n+m)/\sqrt{n^2+nm+m^2}$, which is the number of stick-slip jumps per distance, multiplied by the value of ε_{zz} obtained from the Tomlinson model simulations. The agreement is quite good, considering the simplicity of the model.

Density functional theory calculations of graphene buckling

In order to analyze the Euler buckling of graphene sheets, we perform first-principles calculations based on density functional theory (DFT) and pseudopotentials. The calculations were performed using the Quantum Espresso code[S6]. Wavefunctions

were expanded on a plane-wave basis with an energy cutoff of 50 Ry. For the electron-electron interactions, we use a PBE-GGA exchange-correlation functional[S7] and, for electron-ion interactions, we have use Vanderbilt ultrasoft pseudopotentials[S8]. Within this framework, the calculated lattice constant of the graphene sheet at equilibrium is 2.47 Å. In the out-of-plane direction, we have chosen a vacuum distance of 12 Å. In order to simulate the strain effects, we have built rectangular supercells in which we rescale all coordinates and lattice vectors according to the corresponding strain tensor. We then compare the total energies for two different geometries: one in which the sheet is kept planar throughout the calculation and another in which we start with an initial sinusoidal deformation in the direction of applied stress (Fig. S7a). In both cases, atomic coordinates are fully relaxed. The wavelength of the sinusoidal deformation is chosen to match the supercell length in the corresponding direction; hence we explore different supercell sizes in order to study deformations with different wavelengths. We performed calculations for deformations in both zigzag and armchair directions, with strain values up to 5%. Finally, the k-point sampling of the Brillouin Zone is chosen to be inversely proportional to the supercell length in each direction, with a value of 12 x 12 x 1 (Monkhorst-Pack)[S9] for the primitive lattice of the graphene sheet without deformations.

Starting from a planar graphene sheet at equilibrium, we apply a gradually increasing stepwise compressive strain to the sheet (in absolute values, $\varepsilon = |\Delta L/L|$), along both armchair and zigzag directions. At each step, we monitor the stability of the planar sheet with respect to buckling, as shown schematically in Fig. S7a. Once the strain is larger than a critical value ε_c, that depends on the supercell length L, the sheet buckles. A convenient way to detect the buckling transition is by looking at the stress-strain curve, shown in Fig. S7b for zigzag strain and Fig. S7d for armchair strain, for

several values of unit cell size L along the strain direction. At the critical strain ε_c, the slope of the stress-strain curve changes discontinuously.

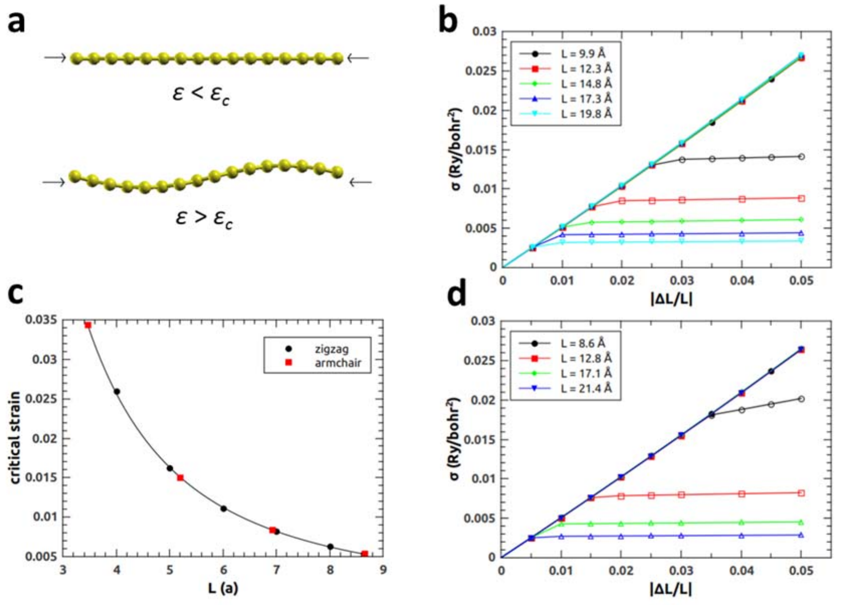

Figure S7. Buckling of graphene. (a) Schematic description of the first-principles calculations of buckling instabilities under strain. Stress strain curves for zigzag (b) and armchair (d) strains for several supercell lengths. Full dots correspond to the planar configuration and open dots to the buckled configuration. The buckling transition occurs where the two curves meet. (c) Critical buckling strain as a function of supercell length, for both zigzag (black circles) and armchair (red squares) directions. The line is a power law fit, which gives approximately an inverse square dependence.

Fig. S7c shows ε_c as a function of L strains along both zigzag and armchair directions. As expected, the critical strain goes to zero in the limit of $L \to \infty$, indicating the critical instability of an infinite and isolated graphene sheet with respect to buckling in the presence of infinitesimally small compressive strains. More interestingly, the ε_c vs L data for armchair (red squares) and zigzag (black circles) directions follow the

same power law curve, indicating that critical buckling strains (and stresses) are isotropic in graphene and thus justifying the use of a single, isotropic critical force F_c in the modified Tomlinson model simulations described in the main text.

Notice that the actual experimental situation differs considerably than that of an isolated and infinite graphene sheet under uniform compressive stress, basically for two reasons: (1) In the case of an AFM scan, the stress is not uniform but it is applied locally at the tip-surface contact point; (2) The sheet is not free, but interacts with a substrate through van der Waals forces. In particular, the latter condition implies that, even in the idealized situation of an infinite sheet, a finite critical stress is needed to buckle a graphene sheet.

Molecular dynamics simulations

In our molecular dynamics simulations, a Tersoff potential[S10] was used to model carbon-carbon interactions and a van der Waals force, as implemented in the LAMMPS software[S11], was used to describe graphene-tip and graphene-substrate interactions. Integration of dynamical equations was carried out considering a constant temperature controlled by a Nose-Hoover thermostat. During all calculations, a 0.1 fs timestep was adopted. Temperatures in a range going from 20K up to 350K were considered and qualitatively equivalent results were obtained for all temperatures.

Movies of the simulations are available online. The 6 movies included in this Supplementary Information show the deformation predicted by our MD model for an AFM tip interacting with a graphene layer deposited on a substrate (not shown). Files named *arm_elastic.mp4* and *zig_elastic.mp4* show the behavior of the AFM tip for a constant normal force (as described in the text) which causes elastic deformations on the tip, while the scanning is through the armchair and zigzag direction respectively. Files

arm_plastic.mp4 and *zig_plastic.mp4*, show what happens when the normal force is one order of magnitude larger, imposing plastic deformations on the tip, while the movies named *arm_vM_plastic.mp4* and *zig_vM_plastic.mp4* show the same simulations but, in this case, the von Mises Stress on the graphene membrane is indicated by a color scale, in which blue regions indicate low, white regions intermediate and red regions indicate high stress values on the structure.

References

S1. Saito, R., Dresselhaus, G., &Dresselhaus, M. S. *Physical Properties of Carbon Nanotubes* (Imperial College Press, London, 1998).

S2. Tomlinson, G. A. Molecular Theory of Friction. *Phil. Mag.* **7**, 905-939 (1929).

S3. Prandtl, L. Ein Gedankenmodell zur Kinetischen Theorie der Festen Körper. *Z. Angew. Math. Mech.* **8**, 85-106 (1928).

S4. Hölscher, H., Schwarz, U. D., Zwäorner, O. & Wiesendanger, R. Consequences of The Stick-Slip Movement For The Scanning Force Microscopy Imaging of Graphite. *Phys. Rev. B* **57**, 2477-2481 (1998).

S5. Allen, M. P., & Tildesley, D. J. *Computer Simulation of Liquids* (Clarendon Press, Oxford, 1989).

S6. Giannozzi P. et al., Quantum Espresso: A Modular And Open-Source Software Project For Quantum Simulations of Materials. *J. Phys.: Condens. Matter* **21**, 395502 (2009).

S7. Perdew, J. P., Burke, K. & Ernzerhof, M. Generalized Gradient Approximation Made Simple. *Phys. Rev. Lett.* 77, 3865-3868 (1996).

S8. Vanderbilt, D. Soft Self-Consistent Pseudopotentials in a Generalized Eigenvalue Formalism. *Phys. Rev. B (R)* **41**, 7892-7895 (1990).

S9. Monkhorst, H. J. & Pack, J. D. Special Points for Brillouin-Zone Integrations. *Phys. Rev. B* **13**, 5188-5192 (1976).

S10. Tersoff, J. Empirical Interatomic Potential for Carbon, with Applications to Amorphous Carbon. *Phys. Rev. Lett.***61**, 2879-2882 (1988).

S11. Plimpton, S. Fast Parallel Algorithms for Short-Range Molecular Dynamics. *J. Comp. Phys.* **117**, 1-19 (1995).
